# Huobahuagen tablet improves renal function in diabetic kidney disease: a real-world retrospective cohort study

**DOI:** 10.3389/fendo.2023.1166880

**Published:** 2023-06-19

**Authors:** Ying Tan, Ruihan Li, Peipei Zhou, Nan Li, Weilong Xu, Xiqiao Zhou, Qianhua Yan, Jiangyi Yu

**Affiliations:** ^1^ Department of Endocrinology, Jiangsu Province Hospital of Chinese Medicine, Affiliated Hospital of Nanjing University of Chinese Medicine, Nanjing, China; ^2^ The First Clinical Medical College, Nanjing University of Chinese Medicine, Nanjing, China

**Keywords:** Huobahuagen tablet, Huangkui capsule, diabetic kidney disease, efficacy, real-world evidence

## Abstract

**Objective:**

We aimed to explore the value of Huobahuagen tablet (HBT) in improving decreased renal function for patients with diabetic kidney disease (DKD) over time.

**Methods:**

This was a single-center, retrospective, real-world study on eligible 122 DKD patients who continued to use HBT + Huangkui capsule (HKC) therapy or HKC therapy without interruption or alteration in Jiangsu Province Hospital of Chinese Medicine from July 2016 to March 2022. The primary observation outcomes included estimated glomerular filtration rate (eGFR) at baseline and 1-, 3-, 6-, 9-, and 12-month follow-up visits and changes in eGFR from baseline (ΔeGFR). Propensity score (PS) and inverse probability treatment weighting (IPTW) were used to control for confounders.

**Results:**

eGFR was significantly higher in the HBT + HKC group than in the HKC alone group at the 6-, 9-, and 12-month follow-up visits (*p* = 0.0448, 0.0002, and 0.0037, respectively), indicating the superiority of HBT + HKC over HBT alone. Furthermore, the ΔeGFR of the HBT + HKC group was significantly higher than that of the HKC alone group at the 6- and 12-month follow-up visits (*p* = 0.0369 and 0.0267, respectively). In the DKD G4 patients, eGFR was higher in the HBT + HKC group at the 1-, 3-, 6-, 9-, and 12-month follow-up visits compared with baseline, with statistically significant differences at the 1-, 3-, and 6- month follow-up visits (*p* = 0.0256, 0.0069, and 0.0252, respectively). The fluctuations in ΔeGFR ranged from 2.54 ± 4.34 to 5.01 ± 5.55 ml/min/1.73 m^2^. Change in the urinary albumin/creatinine ratio from baseline did not exhibit a significant difference between the two groups at any of the follow-up visits (*p* > 0.05 for all). Adverse event incidence was low in both groups.

**Conclusion:**

The findings of this study based on real-world clinical practice indicate that HBT + HKC therapy exhibited better efficacy in improving and protecting renal function with a favorable safety profile than HKC therapy alone. However, further large-scale prospective randomized controlled trials are warranted to confirm these results.

## Introduction

Diabetic kidney disease (DKD), a major microvascular complication of diabetes with an incidence of 30%–40% (1), is the leading cause of end-stage kidney disease and a major social and economic burden worldwide. However, DKD is more difficult to treat than other types of kidney diseases owing to metabolic factors and the effects of metabolic memory (2). The findings of the Irbesartan Diabetic Neuropathy trial (3) and STENO-2 study (4) emphasized that the rigorous control of hyperglycemia and hypertension and use of renin–angiotensin system inhibitors (RASIs) failed not only to control the decrease in the estimated glomerular filtration rate (eGFR) but also to effectively reduce urinary protein levels or delay the decline of renal function in patients with clinical DKD. Although both the CREDENCE (5) and DAPA-CKD (6) studies reported that sodium–glucose cotransporter 2 inhibitors (SGLT2i) exhibited renoprotective effects in addition to lowering blood glucose levels, these drugs can only delay renal function decline but not reverse it. Therefore, the development of novel therapeutic agents that can effectively reverse the decline in renal function in patients with DKD is clinically important.

Traditional Chinese medicine (TCM) has a long history of being commonly used to treat DKD owing to its promising effectiveness in clinical practice because of its multitarget nature (7). Huangkui capsule (HKC) is a TCM comprising the ethanol extract of *Abelmoschus manihot* flowers and has been approved by the Chinese State Food and Drug Administration to treat kidney disease (8). A meta-analysis reported the efficacy and safety of HKC in patients with decreasing serum creatinine (Scr) levels and proteinuria (9). As a representative of traditional Chinese medicine in treating DKD, HKC was selected as the medication of the control group. However, the longest course of DKD treatment with HKC was only 6 months as far as we know, and most research used HKC for treating DKD G1-2 (10, 11). Therefore, for DKD patients with decreased renal function, clinicians prefer to combine HKC with tripterygium glycosides (TGs) extracted from *Tripterygium wilfordii* to enhance renal protection. In 2022, our team’s previous research confirmed that TGs combined with HKC could be more effective in decreasing the Scr levels (11). However, TGs can engender systemic adverse effects with an incidence of 30.75% (12), including gastrointestinal reactions, liver and kidney dysfunction, and reproductive toxicity, particularly in long-term and high-dose clinical applications (13). Thus, the widespread application of TGs for treating chronic kidney diseases is hindered by the narrow therapeutic window owing to severe toxicity.

Huobahua, also known as *Tripterygium hypoglaucum* (Lévl.) Hutch (THH), is a plant native to the southwest region of China that has pharmacological effects similar to those of *T. wilfordii* Hook F (TwHF) with lower toxicity (14). Huobahuagen tablet (HBT) is prepared from the peeled and dried root of THH and can be used to effectively treat chronic kidney disease (CKD). A recent review regarding the clinical applications and pharmacological effects of HBT over the past two decades revealed that HBT exhibited an excellent ability to reduce Scr levels and albuminuria in the treatment of DKD due to its anti-inflammatory and anti-fibrotic properties (15). Moreover, combination treatment with HBT and HKC has been clinically applied as a potential therapeutic regimen for DKD in China. However, to the best of our knowledge, no clinical study to date has focused on the therapeutic efficacy of the combination of HBT and HKC for DKD. To fill this knowledge gap, we conducted a retrospective real-world study (RWS) to evaluate the clinical efficacy and safety of HBT combined with HKC for treating DKD.

## Methods

### Patient selection

**Figure 1:**
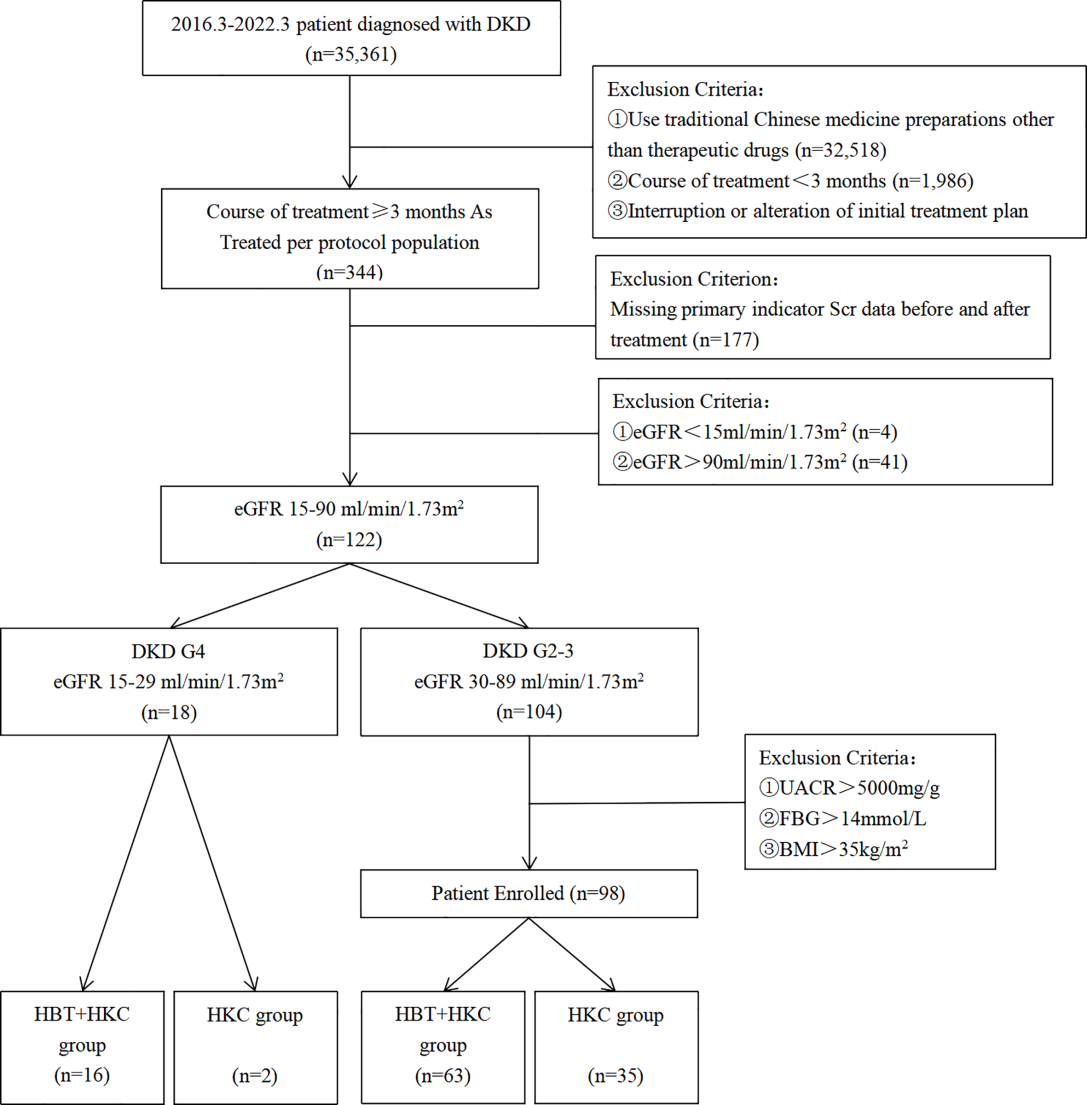
Flowchart of the selection process of the study population.

Overall, 35,361 patients were diagnosed with DKD in the Jiangsu Province Hospital of Chinese Medicine from July 2016 to March 2022 (**Figure 1**). The study cohort included patients with DKD who were treated with HKC or HKC + HBT and fulfilled the inclusion and exclusion criteria. DKD diagnosis was based on the Kidney Disease Outcomes Quality Initiative (KDOQI) clinical practice guidelines (16). According to GFR categories in KDOQI, we described eGFR 30–89 ml/min/1.73 m^2^ as DKD G2-3 and eGFR 15–29 ml/min/1.73 m^2^ as DKD G4 in this paper.

The “as treated” set of 344 patients was selected by excluding patients who used TCM preparations other than the study drugs (n = 32,518), those with a treatment course duration of <3 months (n = 1,986), and those who interrupted or altered the treatment plan during the treatment course (n = 513). Among these 344 patients, those with missing Scr values before or after treatment in patient records (n = 177), those with end-stage kidney disease at baseline (eGFR < 15 ml/min/1.73 m^2^; n = 4), and those without impaired renal function at baseline (eGFR > 90 ml/min/1.73 m^2^; n = 41) were excluded. Additionally, the patients were required to have received a stable dose of RASIs throughout the TCM treatment. The study cohort included 122 patients.

### Study design

This was a retrospective, single-center, RWS study that included primary data regarding patient characteristics, comorbidities, laboratory test results, and medical treatment. This study was initiated by investigators who provided scientific decisions on the study objectives, protocol, conduct, and analysis.

Following the selection of the eligible patients, the ones with mild/moderate renal impairment (DKD G2-3, n = 104) were likely to be assigned HBT + HKC as well as HKC therapy alone, and those with severe renal insufficiency (DKD G4) were more likely to be assigned HBT + HKC therapy in the clinic, as HKC therapy alone might be insufficient based on the evaluation of the collected data and opinion of experts in the field. Therefore, a separate analysis of only HBT + HKC therapy in that group was performed.

According to the TCM treatment, DKD G2-3 patients were divided into the HBT + HKC (n = 63) and HKC alone (n = 35) groups. For DKD G4 patients, only 16 patients who received HBT + HKC were analyzed, and two patients who received HKC alone were excluded due to the lack of representativeness.

### Data collection

Data for the present study were retrospectively collected from the electronic patient records of Jiangsu Province Hospital of Chinese Medicine. Patient records were individually reviewed using a standardized, structured data collection protocol for 1) demographic characteristics and pretreatment events including age, sex, disease course of diabetes, comorbidities, and concomitantly prescribed medicines; 2) physical examination and laboratory results at baseline, including body mass index (BMI), systolic and diastolic blood pressures, fasting blood glucose, total cholesterol, and low-density lipoprotein cholesterol; and 3) primary follow-up efficacy indicators, including Scr levels and urinary albumin/creatinine ratio (UACR). The therapeutic efficacy indices of eGFR and UACR were also calculated. eGFR was calculated using the Chronic Kidney Disease Epidemiology Collaboration (CKD-EPI) equation. Moreover, the DKD stage was inferred accordingly.

### Groups and treatments

In this retrospective observational study, the patients were categorized according to the TCM administered for DKD. To be included in the study, the patients were required to receive stable daily doses of agents that controlled blood glucose, pressure, and lipid levels; have infections; have fluid/electrolyte balance; and have acid–base homeostasis. The following were the medication methods of the different groups:

(1) HKC alone group (HKC): orally treated with HKC five capsules thrice daily (6.45 g/day).
(2) HBT combined with HKC group (HBT + HKC): orally treated with HKC five capsules thrice daily (6.45 g/day) and HBT four capsules thrice daily (2.16 g/day).

### Statistical analysis

Continuous variables were reported as means ± standard deviation for normally distributed data and as medians (25th–75th percentiles) for data with skewed distribution. The normality of data distribution was assessed using Shapiro–Wilk test. Independent and paired *t*-tests were used to assess between- and within-group differences in normally distributed data, and Wilcoxon’s rank-sum and paired rank-sum tests were used to evaluate between- and within-group differences in non-normally distributed data. Categorical variables were described as counts and percentages, reported as n (%), and χ^2^ or Fisher’s exact test was performed for between-group comparisons. All analyses were separately performed in the “as treated” datasets.

In this observational RWS, the two comparison groups exhibited substantial differences in the baseline risk factors, including renal function, age, sex, and risk level, which were primarily introduced by the treatment choice based on the clinical status of patients. Therefore, to control for confounders, propensity score (PS) matching was performed to assess the conditional probability of assignment to a particular treatment given the observed covariates, and inverse probability treatment weighting (IPTW) was applied to balance the two groups. Owing to the contribution to the outcome and the completeness of risk factors, logistic regression including age, sex, baseline eGFR, and risk level as input variables, was applied to calculate the propensity score, and weighting was considered appropriate if the standardized mean difference between the groups was <0.2 following adjustment. After adjustment, no significant differences were noted in the baseline renal function, age, sex, and risk level between the two groups, and the baseline was balanced and comparable.

As the study analysis included data collected as a part of daily practice and not for a specifically designed study protocol, missing key data were inevitable, especially as this non-interventional, long-term study included both inpatients and outpatients. An interpolation strategy was applied to generate corresponding data for the main outcome indicators eGFR and UACR at the time to perform the comparison, which was 1, 3, 6, 9, and 12 months following treatment. According to the reference and development trend, we applied a simple and robust strategy to fill those two: for eGFR, a linear interpolation was used; for UACR, a linear interpolation in logarithmic space was used.

To compare the changes in eGFR and UACR as the endpoints in the between-group analyses and to compare the change in eGFR as the endpoint among different stratifications in the experimental group, logistic regression was used to calculate odds ratios (ORs) with 95% confidence intervals (CIs). Unless otherwise specified, a *p*-value of <0.05 was considered statistically significant, whereas a *p*-value of <0.01 was considered highly significant. IPTW analysis was performed using R statistical software version 4.2.1, and other analyses were performed using SAS 9.4.

## Results

### Patient characteristics

The final analyses included a total of 98 DKD G2-3 patients who fulfilled all the study inclusion and exclusion criteria. Among these, 63 and 35 patients were included in the HBT + HKC (experimental) and HKC alone (control) groups, respectively. Furthermore, a separate study group included 16 DKD G4 patients who were treated with HBT + HKC therapy (**Figure 1**).

The patients who were treated with HKC alone were younger and exhibited shorter disease duration than those treated with HBT + HKC. The balance in the baseline characteristics between the two groups improved following IPTW (standardized differences <0.2; **Table 1**). Age, sex, disease course, and eGFR were more balanced and demonstrated no statistical differences between the groups; furthermore, after matching, BMI, systolic blood pressure, fasting blood glucose, UACR, total cholesterol, and low-density lipoprotein cholesterol were not significantly different between the two groups.

**Table 1:** Baseline demographic and clinical characteristics of the DKD G2-3 patients.

Characteristics		Before IPTW			After IPTW		
		HBT + HKC (n = 63)	HKC (n = 35)	*p*-Value	HBT + HKC (n = 63)	HKC (n = 35)	*p*-Value
Age (years)		55.40 ± 10.15	60.60 ± 8.49	0.0117	57.00 (50.00, 63.00)	56.00 (51.00, 66.00)	0.4138
Sex	Male (%)	51 (81.0)	26 (74.3)	0.4409	77 (80.6)	73 (79.0)	0.7967
	Female (%)	12 (19.0)	9 (25.7)		19 (19.4)	19 (21.0)	
Duration of diabetes (years)		10.00 (9.00, 16.00)	16.00 (9.50, 21.50)	0.0579	10.00 (9.00, 20.00)	13.00 (9.00, 18.00)	0.6955
BMI (kg/m^2^)		25.65 ± 2.99	25.11 ± 2.41	0.5123	24.90 (23.01, 26.75)	25.14 (24.62, 27.53)	0.3653
Systolic BP (mmHg)		142.50 (132.50, 165.00)	138.50 (129.50, 158.00)	0.5343	150.00 (135.00, 180.00)	133.00 (130.00, 143.00)	0.0739
Diastolic BP (mmHg)		91.25 ± 6.29	78.70 ± 7.85	0.0067	90.00 (90.00, 100.00)	78.00 (77.00, 85.00)	0.0021
FBG (mmol/L)		6.99 (6.07, 8.69)	6.53 (5.85, 7.60)	0.2733	7.48 (6.14, 9.27)	6.81 (5.85, 8.72)	0.1925
eGFR (ml/min/1.73 m^2^)		55.40 (40.07, 70.18)	57.68 (44.07, 70.89)	0.5832	59.09 (39.29, 70.18)	51.72 (44.07, 70.89)	0.9542
UACR (mg/g)		1,228.00 (343.00, 1,799.00)	518.00 (319.00, 1,056.00)	0.3209	808.00 (213.00, 1,799.00)	812.00 (412.00, 2,176.00)	0.1326
TC (mmol/L)		4.89 ± 1.38	4.70 ± 1.07	0.7022	4.86 (4.68, 5.69)	4.02 (2.86, 6.05)	0.5685
LDL-C (mmol/L)		3.21 (2.13, 3.72)	2.63 (2.14, 3.73)	0.926	3.56 (2.41, 3.71)	2.24 (2.19, 3.77)	0.6837
Hypertension (%)		50 (79.4)	9 (25.7)	<0.0001	77.5 (80.7)	23.2 (25.2)	<0.0001

BMI, body mass index; BP, blood pressure; FBG, fasting blood glucose; eGFR, estimated glomerular filtration rate; UACR, urinary albumin/creatinine ratio; TC, total cholesterol; LDL-C, low-density lipoprotein cholesterol; IPTW, inverse probability treatment weighting; DKD, diabetic kidney disease.

In the group of patients with DKD G4, the mean age was 63.63 ± 7.19 years, and men accounted for 62.5%. The mean course of the disease was 17.90 ± 5.57 years, the mean BMI was 24.91 ± 3.67 kg/m^2^, the mean eGFR at baseline was 21.70 ± 4.50 ml/min/1.73 m^2^, and the mean UACR was 3,062.54 ± 2,579.54 mg/g (**Table 2**).

**Table 2:** Baseline demographic and clinical characteristics of the DKD G4 patients.

Characteristics		HBT + HKC (n = 16)
Age (years)		63.63 ± 7.19
Sex	Male (%)	10 (62.50)
	Female (%)	6 (37.50)
Duration of diabetes (years)		17.90 ± 5.57
BMI (kg/m^2^)		24.91 ± 3.67
Systolic BP (mmHg)		158.00 (124.00, 159.00)
Diastolic BP (mmHg)		77.67 ± 14.50
FBG (mmol/L)		6.06 ± 1.40
eGFR (ml/min/1.73 m^2^)		21.70 ± 4.50
UACR (mg/g)		3,778.29 ± 2,485.81
TC (mmol/L)		7.45 ± 2.39
LDL-C (mmol/L)		4.88 ± 1.83
Hypertension (%)		14 (87.50)

BMI, body mass index; BP, blood pressure; FBG, fasting blood-glucose; eGFR, estimated glomerular filtration rate; UACR, urinary albumin/creatinine ratio; TC, total cholesterol; LDL-C, low-density lipoprotein cholesterol; DKD, diabetic kidney disease.

### Clinical outcomes

#### Comparing the efficacy of HBT + HKC and HKC alone

The eGFR and UACR were calculated at baseline and 1-, 3-, 6-, 9-, and 12-month follow-up visits. Subsequently, the cohorts were weighted using IPTW, the statistical description of each visit was summarized, and changes in the variables from the baseline were calculated. Next, between-group differences in absolute values and changes from baseline were evaluated using Wilcoxon’s rank-sum test.

**Figure 2:**
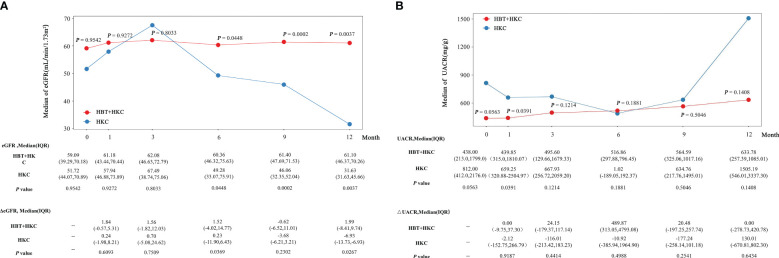
Effectiveness of HBT + HKC versus HKC alone on eGFR and UACR in DKD G2-3 patients over time. **(A)** The line plot shows the eGFR at specific time points. The eGFR, ΔeGFR, and *p*-values are listed below the line plot. **(B)** The line plot shows the UACR at specific time points. The UACR, ΔUACR, and *p*-values are listed below the line plot. *p*-Values were between-group statistical results at baseline and 1-, 3-, 6-, 9-, and 12-month time points. HBT, Huobahuagen tablet; HKC, Huangkui capsule; eGFR, estimated glomerular filtration rate; UACR, urinary albumin/creatinine ratio; DKD, diabetic kidney disease.

The HBT + HKC group exhibited a stable trend of eGFR, the primary therapeutic index, after the baseline. Additionally, eGFR increased at the 1-, 3-, 6-, 9-, and 12-month follow-up visits compared with the baseline value (**Figure 2A**). The HKC alone group exhibited an upward trend from the 1- to 3-month follow-up visits, followed by a downward trend till the 12-month follow-up visit. The baseline eGFR did not significantly differ between the two groups (*p* = 0.954). However, eGFR was significantly higher in the HBT + HKC group than in the HKC alone group at the 6-, 9-, and 12-month follow-up visits (*p* = 0.0448, 0.0002, and 0.0037, respectively), whereas no significant difference was noted at the 1- and 3-month follow-up visits (*p* > 0.05). Additional analyses included comparing the change in eGFR (ΔeGFR) between the HBT + HKC and HKC alone groups and the baseline; ΔeGFR was calculated for each visit after the baseline. Briefly, ΔeGFR was significantly higher in the HBT + HKC group than in the HKC alone group at the 6- and 12-month follow-up visits (*p* = 0.0369 and 0.0267, respectively). Furthermore, median ΔeGFR was higher in the HBT + HKC group than in the HKC alone group at the 1-, 3-, and 9-month follow-up visits, although the differences were not statistically significant (*p* > 0.05).

In the HBT + HKC group, UACR exhibited a slow upward trend after the baseline, albeit without statistical significance (**Figure 2B**). Conversely, in the HKC alone group, UACR followed a downward trend from the 1- to 6-month follow-up visits, subsequently showing an upward trend from the 6- to 12-month follow-up visits; however, these changes were not statistically significant. Although no statistically significant difference was observed in the baseline UACR values of the two groups (*p* > 0.05), at the 1-month follow-up visit, UACR was significantly lower in the HBT + HKC group than in the HKC alone group (*p* = 0.0391). No significant differences in UACR values were observed between the two groups at the 3-, 6-, 9-, and 12-month follow-up visits (*p* > 0.05); however, the median UACR was lower in the HBT + HKC group than in the HKC alone group at the 3-, 9-, and 12-month follow-up visits. The change in UACR from the baseline (ΔUACR) was calculated for each visit, and differences in ΔUACR between the two groups were compared, revealing that the two groups did not exhibit significant differences in ΔUACR at any of the visits (*p* > 0.05).

#### Subgroup and sensitivity analyses

**Figure 3:**
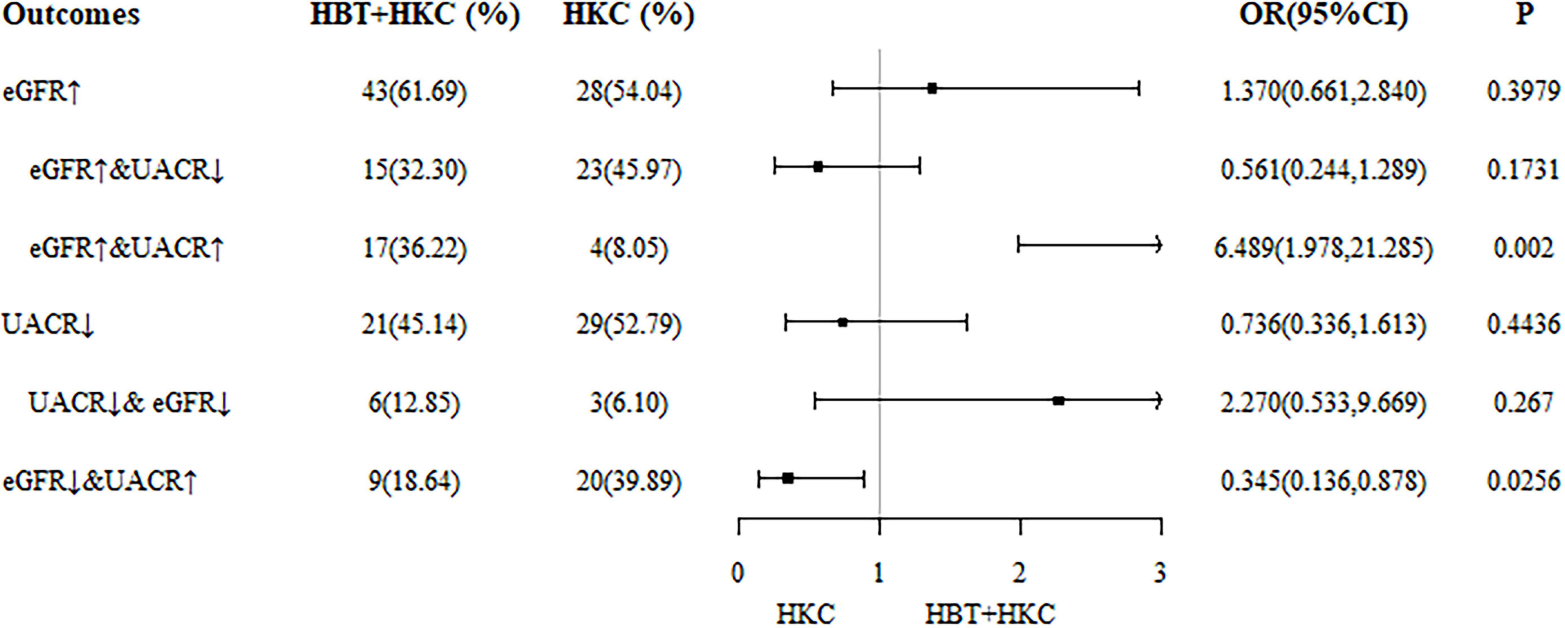
Forest plot of differences in eGFR and UACR improvement rates at 6 months. eGFR, estimated glomerular filtration rate; UACR, urinary albumin/creatinine ratio.

As the main therapeutic efficacy indicators, eGFR and ΔeGFR exhibited statistically significant superiority in the HBT + HKC group compared with the HKC alone group at 6 months. Furthermore, the data at 6 months were complete as dropout occurred at the 9th and 12th months; therefore, the results at 6 months were selected for comparison between the two groups. The differences in the eGFR and UACR improvement rates were compared, and the corresponding forest plots were drawn (**Figure 3**).

The study cohort was further categorized into the following six subgroups according to the outcome: 1) increasing eGFR, 2) increasing eGFR and decreasing UACR, 3) increasing eGFR and UACR, 4) decreasing UACR decreasing, 5) decreasing eGFR and UACR, and 6) decreasing eGFR and increasing UACR. With the use of IPTW data, the number and percentage of each case at 6 months were determined. Logistic regression analyses revealed that in subgroup 3 (increasing eGFR and UACR), the proportion of patients treated with HBT + HKC was significantly higher than that of the patients treated with HKC alone (36.22% versus 8.05%; OR 6.489, 95% CI 1.978–21.285; *p* = 0.002), indicating that the HBT + HKC group had more patients with improved eGFR but not with improved UACR compared with the HKC alone group. In subgroup 6 (decreasing eGFR and increasing UACR), the proportion of patients treated with HBT + HKC was significantly lower than that of the patients treated with HKC alone (18.64% versus 39.89%; OR 0.345, 95% CI 0.136–0.878; *p* = 0.0256), indicating that the HKC alone group had more patients with worsening eGFR and UACR, suggesting that the inefficiency of both eGFR and UACR was significantly higher in the control group.

#### Efficacy of HBT + HKC therapy in DKD G4 patients

**Figure 4:**
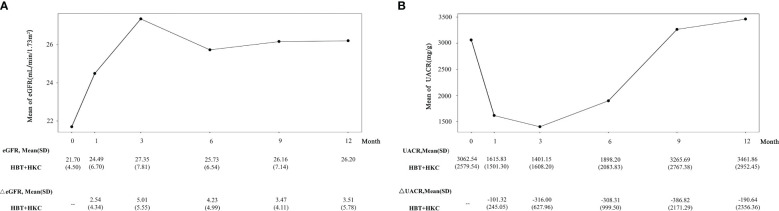
Effect of HBT + HKC therapy on eGFR and UACR over time. **(A)** The line plot shows the eGFR at specific time points. The eGFR and ΔeGFR values are listed below the line plot. **(B)** The line plot shows the UACR at specific time points. The UACR and ΔUACR values are listed below the line plot. HBT, Huobahuagen tablet; HKC, Huangkui capsule; eGFR, estimated glomerular filtration rate; UACR, urinary albumin/creatinine ratio.

In patients with DKD G4, an increasing trend in eGFR (**Figure 4A**) with fluctuations was observed from the baseline to the 12-month follow-up visit; the eGFR values at all the follow-up visits were higher than the baseline value. Moreover, eGFR was significantly higher at the 1-, 3-, and 6-month follow-up visits compared with the baseline (*p* = 0.0256, 0.0069, and 0.0252, respectively). The fluctuation in ΔeGFR ranged from 2.54 ± 4.34 to 5.01 ± 5.55 ml/min/1.7 m^2^.

In these patients, UACR exhibited an initial decreasing trend, with a subsequent increase. The best improvement (i.e., reduction in UACR) was noted at the 3-month follow-up visit, slightly higher than the baseline at 9 and 12 months, and no statistical significance was noted in the within-group comparison (*p* > 0.05) (**Figure 4B**). For ΔUACR, from 1 to 9 months, the reduced value from the baseline almost gradually increased. The fluctuation in ΔUACR ranged from −386.82 ± 2,171.29 to −101.32 ± 245.05 mg/g.

### Adverse events

In the study cohort, two of the 63 patients in the HBT + HKC group developed adverse events, including abdominal discomfort and constipation in each; the incidence of adverse events was 3.2%. Among the 35 patients in the HKC group, one patient developed abdominal discomfort, and the incidence of adverse events was 2.9%. The incidence of adverse events was comparable between the HBT + HKC and HKC alone groups (*p* > 0.05). No adverse events were reported in the DKD G4 patients who received HBT + HKC therapy. All adverse events were mild and resolved after symptomatic treatment.

## Discussion

In the present study involving patients with DKD, HBT + HKC therapy exhibited better efficacy than the HKC therapy alone in improving renal function in DKD G2-3 patients, with the observed efficacy becoming more evident with increasing therapy duration. At 12 months, under similar baseline conditions, the eGFR of the HBT + HKC group was stable at 61.1 ml/min/1.73 m^2^, whereas that of the HKC alone group decreased to 31.63 ml/min/1.73 m^2^; the difference in the eGFR between the two groups was nearly twofold. For DKD patients with severe renal insufficiency (DKD G4), the eGFR exhibited an increasing trend following treatment with HBT + HKC. After 3–12 months of continuous treatment, the eGFR increased from 21.70 (4.50) to 27.35 (7.81) ml/min/1.73 m^2^, indicating that the HBT + HKC therapy was associated with a trend toward improvement in renal function to some extent. In addition, the clinical effectiveness of the HBT + HKC therapy in this real-world study was unaccompanied by any significant adverse reactions.

eGFR is the most common prognostic indicator used for the renal function of DKD patients in clinical trials and practice (17, 18). Because eGFR can be affected by some interference factors (19), continuous monitoring of long-term and multi-time points is more meaningful. However, many clinical trials of TCM therapy for DKD were only for the observation of therapeutic indicators at the time point before and after treatment. Similar to previous longitudinal DKD studies (20, 21), our study also chose multiple time points, allowing for a more direct and robust observation of change in renal function and UACR over time, which included baseline and 1-, 3-, 6-, 9-, and 12-month time points. Meanwhile, there were some differences between our study and previous clinical studies on the treatment of DKD with traditional Chinese medicine. 1) Selection of the control group: most clinical trials used angiotensin-converting enzyme inhibitors (ACEIs)/angiotensin receptor blockers (ARBs) as the control group, and the treatment group received the combined therapy of Chinese herbal medicine and ACEI/ARB (22–24). Contrary to previous trials, our study selected HKC as the drug of the control group based on a stable dose of ARB/ACEI treatment. Systematic review and meta-analysis have demonstrated that HKC may treat DKD effectively (9). In 2022, a randomized, double-blind, parallel-controlled multicenter clinical trial further confirmed that HKC combined with irbesartan could exert a greater beneficial effect on DKD than the two alone (25). Therefore, the results of HBT on improving renal function in our study were obtained based on comparison with a more effective control group. 2) The course of treatment: the course of previous clinical studies related to HKC or TwHF was mostly limited to 6 months, while the treatment period of our study was extended to 1 year, which not only filled the clinical gap related to HBT or HKC but also made its efficacy and safety in improving renal function more convincing. In addition, in the real-world clinical setting, many DKD patients go to the hospital only when they find a significant decline in renal function or overt proteinuria, which can be called the “silent crowd effect” (26, 27). For such DKD patients with decreased renal function, the clinical efficacy of HKC alone is unknown. Our study found that when HKC alone was continuously used for 6–12 months, eGFR showed a significant downward trend compared to baseline, and the clinical efficacy was far less than that of HBT + HKC therapy, which might explain why so few DKD patients were using HKC alone in DKD G4 and emphasize the necessity and importance of HBT for DKD patients.

The pathogenesis of DKD is complex, and many factors are involved, such as metabolic disorders, hemodynamic abnormalities, oxidative stress, inflammatory response, and epigenetics (28). The essence of DKD can be expressed as inflammation-induced excessive tissue self-repair and subsequent chronic fibrosis, which ultimately leads to loss of renal function (29). Several epigenetic modifications, extensively studied the mechanism of metabolic memory, are involved in the progression of inflammation and fibrogenesis (2, 30). However, ACEI/ARB, which is widely used clinically, does not have anti-inflammatory and anti-fibrotic effects, which to some extent limit the improvement of renal function. TCM has both anti-inflammatory and anti-fibrotic effects by reversing epigenetic modification (22). HBT in this study, similar to HKC and TwHF, can act on inflammatory response and fibrosis process. Pharmacological studies have confirmed that HBT is an effective and low-toxicity Chinese patent medicine, which contains large amounts of flavonoids, such as (+)-catechin and l-epicatechin, and low quantities of terpenoids (14, 31). Because of the active ingredients, HBT can reduce inflammation, combat oxidative stress, protect renal tubular epithelial cells, and inhibit renal fibrosis with low toxicity (14). In addition, terpenoids of the extracts of *T. wilfordii* plants, such as triptolide and celastrol, have high toxic side effects (32, 33), while flavonoid components are relatively safe, which may explain the low toxic side effects of HBT in clinical practice. Notably, epicatechin (EC), as the most abundant natural flavonoid in THH, has been shown to have an immediate protective effect on DKD (34), which cannot be found in HKC and TwHF. EC can not only directly inhibit inflammatory factors (TNF-α, iNOS, and IL-6) but also prevent activation of the TLR4-NF-κB pathway and NOX-dependent oxidant production to reduce proinflammatory factor expression (34, 35). DCCT/EDIC and UKPDS follow-up studies have found that patients with poor early blood sugar control cannot prevent the development of microvascular complications even if they intensify blood sugar reduction later, a phenomenon academically called the adverse “metabolic memory” effect, which is considered an essential reason for the progression of renal fibrosis (2, 36). Studies have confirmed that various post-transcriptional/translation modifications (such as acetylation and microRNA) in a high blood sugar environment have the nature of “metabolic memory” and can be inherited. The resulting epigenetic changes can cause related gene expression disorders, leading to sustained excessive secretion of inflammation and fibrosis factors, resulting in chronic “inflammation-fibrosis” damage to organs (2). EC has been shown to induce epigenetic changes by regulating the levels of histone acetyltransferases (HATs) and histone deacetylase 4 (HDAC4), decreasing H3K9 acetylation and H3K4 dimethylation and increasing H3K9 dimethylation triggered by high glucose (37), thereby indirectly downregulating the TGF-β1 pathway to reduce the deposition of extracellular matrix (38), which play an important role in DKD treatment. Therefore, HBT, in which the primary active component is EC, may regulate epigenetics (acetylation modification) and block the adverse “metabolic memory” effect mediated DKD renal fibrosis, which may provide a breakthrough direction for DKD prevention and treatment. However, further basic experiments are needed to verify this hypothesis. Thus, this study focused on the clinical effect of HBT on DKD.

We found that for DKD G2-3 patients, HKC decreased UACR within 6 months, which was consistent with previous literature (25). UACR of the HBT + HKC group exhibited a gentler trend compared with that of the HKC group at 12 months; however, no significant difference in efficacy was observed. For DKD G4 patients, UACR demonstrated a significant downward trend within 6 months of HBT + HKC therapy. However, no remarkable and stable effect on UACR reduction was noted in the HBT + HKC group, which was slightly inconsistent with our expectations. One plausible reason for this finding is that the observed UACR is easily affected by several factors, such as dietary protein intake, physical exercise, metabolic disorder, and fever, and the urine protein level itself cannot completely reflect the degree of renal function (1, 18). Of course, the possibility that some patient characteristics may have affected therapeutic outcomes cannot be completely ruled out, as shown in the subgroup analyses. However, it needs a larger subgroup sample size to confirm.

The present study has several strengths. First, to the best of our knowledge, this is the first clinical study to compare the efficacy of HBT combined with HKC to that of HKC alone in patients with DKD. Our analyses indicated that the efficacy of the combination therapy might be superior to that of HKC alone in patients with DKD and decreased eGFR. Additionally, the observed efficacy might become more evident after 6–12 months of treatment, without major side effects. Furthermore, HBT + HKC therapy represents a potential treatment option that may increase eGFR and truly achieve the effect of reversing renal function for DKD G4 patients. Second, this is also the first RWS on the treatment of DKD with HBT; we aimed to achieve results that are more closely related to real-world clinical settings. Therefore, based on clinical authenticity, our findings might provide a reference for follow-up randomized controlled studies. Finally, we used rigorous eligibility criteria; that is, we only included patients with DKD who had three consecutive months of treatment with the study drugs following their first drug exposure. The “as treated” patient cohort ensured that all the patients had sufficient data depth.

The present study has several limitations. First, our study was a retrospective RWS, so the data were based on the patients’ detection at that time, which indicated that we were unable to capture data on relevant indicators of inflammation and fibrosis if undetected to support the mechanism. In the future, we will conduct prospective research on HBT to confirm the mechanism. Second, our strict control standards for the use of HBT or HKC resulted in a relatively small sample size after the screening. Meanwhile, many DKD patients who visited the hospital progressed to the middle and late stages due to therapeutic inertia, which resulted in fewer patients using HKC alone. Additionally, the results regarding DKD G4 patients should be interpreted with caution owing to the small sample size. Prospective, randomized, double-blind studies are needed in the future to validate the results.

## Conclusion

In conclusion, this is the first RWS to evaluate the efficacy and safety of HBT in combination with HKC for the treatment of DKD. The current study on HBT is limited, and sufficient attention has not been given to the relationship between ingredients, pharmacological activities, and toxicity. This study is a good attempt at real-world research on TCM.

## Data availability statement

The original contributions presented in the study are included in the article/supplementary material. Further inquiries can be directed to the corresponding authors.

## Ethics statement

Ethical review and approval was not required for the study on human participants in accordance with the local legislation and institutional requirements. The patients/participants provided their written informed consent to participate in this study. Written informed consent was obtained from the individual(s) for the publication of any potentially identifiable images or data included in this article.

## Author contributions

YT and JY contributed to the concept and design of this study. YT and QY carried out the clinical studies, collected data, completed statistical analyses, and drafted and revised the manuscript. RL and PZ participated in the data collection. JY, NL and WX validated data and gave some suggestions. JY and XZ critically revised the manuscript and gave many suggestions. All authors contributed to the article and approved the submitted version.
